# LUHMES-derived 3D organoids as an enhanced platform for modelling dopaminergic neurodegeneration in Parkinson's disease

**DOI:** 10.3389/fmolb.2026.1807358

**Published:** 2026-07-28

**Authors:** Cameron Keighron, Jose C. Casa-Martinez, Serena Barral, Leo R. Quinlan

**Affiliations:** 1 Discipline of Physiology, Cellular Physiology Research Lab, School of Pharmacy and Medical Sciences, University of Galway, Galway, Ireland; 2 Developmental Neurosciences, Zayed Centre for Research, UCL Great Ormond Street Institute of Child Health, University College London, London, United Kingdom; 3 CÚRAM SFI Research Centre for Medical Devices, University of Galway, Galway, Ireland

**Keywords:** 3D cell culture, disease modelling, LUHMES, mitochondrial dysfunction, neurodegeneration, organoids, Parkinson’s disease

## Abstract

**Background:**

Parkinson’s disease (PD) research traditionally relies on animal models and two-dimensional (2D) culture models. These models fail to recapitulate the complex cellular architecture and network interactions that are possible with three-dimensional (3D) organoid models. While 3D models allow improved cell-cell interactions, spatial organization, and metabolic microenvironments, however, their utility for modelling dopaminergic neurodegeneration remains underexplored.

**Methods:**

We developed and characterized 3D organoids from LUHMES cells which are human embryonic neuronal precursor cells and a well-established human dopaminergic neuronal cell line. We directly compare their responses to the mitochondrial toxin MPP+ against conventional 2D cultures. Functional readouts included cell viability, ATP production, dopaminergic marker expression (tyrosine hydroxylase, MAP2, β-III tubulin), reactive oxygen species (ROS) generation, electrophysiological activity via multi-electrode arrays (MEA), and calcium signalling dynamics.

**Results:**

3D LUHMES organoids had 9-fold higher synapsin expression compared to 2D cultures, indicating enhanced synaptic maturity and network complexity. Following acute MPP+ exposure (0.25 mM, 24 h), 3D organoids were significantly more sensitive than 2D cultures, with greater reductions in cell viability (35% vs. 31%), ATP production (36% vs. 31%), and dopaminergic marker expression (TH: 60% reduction in both; MAP2: 70% vs. 54%; TUJ1: 62% vs. 20%). ROS production increased uniformly in 3D organoids (85% positive cells) compared to heterogeneous accumulation in 2D cultures (78% positive cells). Functional assessments revealed that 3D organoids displayed higher baseline electrophysiological activity that was sensitive to impairment following MPP+ treatment, spike amplitude, and calcium signalling responses to various stimuli (ATP, glutamate, GABA).

**Conclusion:**

LUHMES-derived 3D organoids demonstrate greater physiological relevance for modelling PD-related dopaminergic neurodegeneration than 2D cultures. The enhanced sensitivity to mitochondrial toxins, combined with more sophisticated network architecture and functional properties, suggests this model is a valuable platform for mechanistic studies of neurodegeneration and preclinical drug screening. These findings also support the broader adoption of 3D culture systems in neurodegenerative disease research.

## Graphical Abstract

**Figure:**
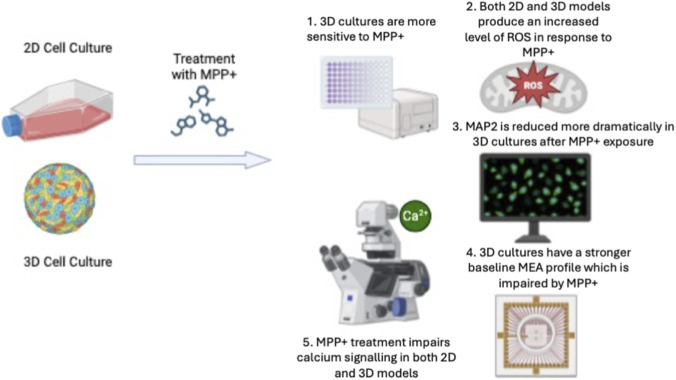

## 1 Introduction

Parkinson’s disease (PD) is the second most common neurodegenerative disorder worldwide, characterised by the progressive loss of dopaminergic neurons in the substantia nigra pars compacta and the accumulation of α-synuclein aggregates known as Lewy bodies (Luo et al., 2024). Understanding the molecular mechanisms underlying dopaminergic neurodegeneration is critical for developing disease-modifying therapies, yet current research models present significant limitations. Animal models, including transgenic mice expressing mutant forms of α-synuclein, LRRK2, or Parkin and toxin-based models using MPP^+^, for example, have provided valuable insights into motor deficits and metabolic deficits associated with neurodegeneration. However, these models come with limitations, both scientific and ethical, that prompt us to question whether they remain the most optimal tools for studying PD (Blesa, Phani, Jackson-Lewis and Przedborski, 2012; Dawson et al., 2010). Traditional two-dimensional (2D) cell culture models offer controlled, reproducible systems for studying cellular mechanisms but lack the architectural complexity, cell-cell interactions, and microenvironmental gradients essential for modelling neurodegeneration (Centeno, Cimarosti and Bithell, 2018; Festing and Wilkinson, 2007).

Induced pluripotent stem cell (iPSC) models enable the generation of patient-specific dopaminergic neurons, capturing the unique genetic background of the patient, including mutationsin LRRK2, PINK1, and SNCA that drive familial forms of PD (Bolognin et al., 2019; Ke, Chong and Su, 2019). iPSC-derived neurons can be used to study early cellular phenotypes such as mitochondrial dysfunction, impaired autophagy, and α-synuclein aggregations, which are often difficult to replicate in traditional models, however, they require lengthy differentiation protocols, exhibit batch-to-batch variability, and often display incomplete maturation in standard 2D formats (Monzio Compagnoni et al., 2018; Zhang, Hu, Shang and Qi, 2020). LUHMES (Lund human mesencephalic) cells represent an alternative human-derived model system specifically designed for dopaminergic neuron research (Lotharius et al., 2005). Originally derived from ventral mesencephalic tissue, they are embryonic neural precursor cells that are conditionally immortalised. LUHMES cells rapidly differentiate into post-mitotic dopaminergic neurons that express tyrosine hydroxylase (TH), develop extensive neuritic networks, and demonstrate functional neurotransmitter release (Lotharius et al., 2002; Scholz et al., 2011) Their. consistent dopaminergic phenotype, rapid differentiation kinetics (4–6 days), and reproducibility have established LUHMES as a valuable model for mechanistic toxicology studies and drug screening (Zhang, Yin and Zhang, 2014). However, LUHMES cells have been almost exclusively utilized in 2D monolayer configurations, limiting their ability to model the three-dimensional cellular interactions and network complexity relevant to neurodegeneration.

In recent years, researchers have made significant advancements in ways we can model disease, from 3D organoids or assembloids. 3D culture models such as brain organoids and assembloids have emerged as promising platforms for specifically modelling the structural and functional complexity of human neuronal networks (Kim and Lee, 2025; Pagliaro, Artegiani and Hendriks, 2025; Paşca, 2018). Recent advances have demonstrated that 3D neural cultures exhibit enhanced metabolic complexity, more physiologically relevant responses to oxidative stress, and improved drug screening predictability compared to 2D systems (Oyefeso, Muotri, Wilson and Pecaut, 2021). Furthermore, assembloid approaches pioneered by the Pasca lab, combining distinct brain regions or cell types enable investigation of circuit-level interactions implicated in PD pathogenesis, such as striatal-nigral connectivity and glial-neuronal crosstalk (Kim and Lee, 2025; Kim et al., 2025; Onesto, Kim and Pasca, 2024; Paşca, 2018). Despite these advances, three-dimensional organoid models specifically employing LUHMES cells for PD research remain underexplored. The rapid differentiation and consistent dopaminergic phenotype of LUHMES cells could address key limitations of iPSC-derived organoids, including lengthy culture periods (months) and variability, while the 3D architecture could overcome the spatial and functional constraints of conventional 2D LUHMES cultures. Such a model could provide an accessible, reproducible platform for studying dopaminergic neurodegeneration with enhanced physiological relevance.

Here, we present data characterising 3D LUHMES organoids and systematically compared their properties to conventional 2D LUHMES cultures. We hypothesised that 3D LUHMES organoids would exhibit enhanced neuronal maturation, more complex network architecture, and greater sensitivity to PD-relevant neurotoxic insults compared to 2D cultures. To test this, we employed the mitochondrial Complex I inhibitor MPP+ (1-methyl-4-phenylpyridinium), a well-established neurotoxin that selectively targets dopaminergic neurons and models key features of PD pathology, including mitochondrial dysfunction, oxidative stress, and cell death (Vila and Przedborski, 2003). We characterised both model systems using complementary approaches spanning cell viability, molecular markers of dopaminergic identity, oxidative stress responses, and functional assessments of electrophysiological activity and calcium signalling. Our findings demonstrate that LUHMES-derived 3D organoids offer significant advantages over 2D cultures for modelling dopaminergic neurodegeneration and provide a valuable platform for mechanistic investigation and therapeutic development in PD research.

## 2 Materials and methods

### 2.1 Cell culture

LUHMES (kindly gifted from Professor Marcel Leist, Leist Lab, Universitätsstraße 10, 78464 Konstanz, Germany) cells were plated on T-75 culture flasks pre-coated with 50 μg/mL poly-L-ornithine (P4957, PLO; Sigma-Aldrich) and 1 μg/mL fibronectin from human plasma (F0895, Sigma-Aldrich). Cells were maintained growth medium of advanced DMEM/F12 supplemented with 1% N-2 supplement (Gibco), 2 mM L glutamine, and 1% penicillin-streptomycin in a 37 °C, 5% CO_2_, humidified incubator. The medium was replenished every 3 days. LUHMES cells were differentiated based on previously reported protocols (Harischandra et al., 2020; Zhang et al., 2014). LUHMES cells were exposed to a differentiation medium consisting of advanced DMEM/F12, 2 mM L-glutamine, 1 mM dibutyryl-cAMP (dbcAMP; (Sigma-Aldrich) 1 μg/mL tetracycline hydrochloride (Sigma-Aldrich), and 1% penicillin-streptomycin for 4 days.

For 3D organoids, cells were plated on T-75 culture flasks pre-coated with 50 μg/mL poly-L-ornithine (PLO; Sigma-Aldrich) and 1 μg/mL fibronectin from human plasma (Sigma-Aldrich). Cells were maintained growth medium of advanced DMEM/F12 supplemented with 1% N-2 supplement (Gibco), 2 mM L glutamine, and 1% penicillin-streptomycin in a 37 °C, 5% CO_2_, humidified incubator. The medium was replenished every 3 days. LUHMES cells were seeded into ultra-low attachment 96 well plates and placed on a shaker for 14 min. After this, they were placed into the incubator and allowed to proliferate for 8 days, with media changes every second day. On day 8, the media was removed and 20ul of Geltrex (A14133022, Thermo) was added to each well. LUHMES organoids were then exposed to a differentiation medium consisting of advanced DMEM/F12, 2 mM L-glutamine, 1 mM dibutyryl-cAMP (dbcAMP; (Sigma-Aldrich) GDNF, 1 μg/mL tetracycline hydrochloride (Sigma-Aldrich), and 1% penicillin-streptomycin for 4 days (moved to 6-well ultra-low attachment plates on day 9).

To induce disease-like phenotypes, cells were treated at a well referenced concentration of 0.25 mM MPP+ for 24 h.

### 2.2 Cell viability assay

Briefly, cells were seeded in 96-well plates at 40 × 10^3^ cells per well directly into differentiation media. Once differentiation was complete, MPP+ treatments were added for a treatment period of 24 h. After 24 h and to measure viability, culture medium was replaced with phosphate-buffered solution containing 10% (*v*/*v*) Alamar blue, and cultures were incubated for 3 h at 37 °C in humidified 95% air/5% CO_2_. The plates were visually inspected for a colour change from blue to pink in control wells. Alamar blue fluorescence was measured in a Hidex automated plate-reading fluorometer, with excitation at 530 nm and emission at 590 nm. Organoids were individually placed into wells of a 96-well plate where the excess media was carefully removed and replaced with phosphate-buffered solution containing 10% (*v*/*v*) Alamar blue and incubated for 3 h at 37 °C in humidified 95% air/5% CO_2_. The plates were visually inspected for a colour change from blue to pink in control wells. Alamar blue fluorescence was measured in a Hidex automated plate-reading fluorometer, with excitation at 530 nm and emission at 590 nm. These measurements were repeated for technical and biological replicates.

### 2.3 ATP production

LUHMES cells were seeded in a 96-well plate and incubated under standard differentiation culture conditions to allow for adherence and maturity. Cell Glo Titer 2.0 (Promega) reagents were prepared by mixing luciferase, luciferin, and buffer components to produce a luminescent signal proportional to ATP levels. The mixed reagent (4 μL) was added to each well, and the plate was shaken briefly to ensure mixing. Luminescence was measured using a plate reader, with signal intensity directly correlating to cell viability. After this, the plate was read at 30 min, 6 h, 24 h, 48 h, and 72 h on a Hidex Plate reader.

In 3D culture, after maturation, organoids were individually placed back into a ultra-low attachment 96 well plate with differentiation medium and Cell Glo Titer 2.0 (Promega) reagents. They were prepared by mixing the reagents as above. The mixed reagent (4 μL) was added to each well, and the plate was briefly placed on a shaker to ensure mixing. Luminescence was measured using a plate reader, with signal intensity directly correlating to ATP production. After this, the plate was read at 30 min, 6 h, 24 h, 48 h, and 72 h on a Hidex Plate reader.

### 2.4 Fluorescence microscopy

To confirm the presence of dopaminergic characteristics, cells were seeded in six healthy plates (250 × 10^3^ cells per well), and their respective differentiation protocols were completed. Cells were treated with 0.25 mM MPP+ alongside untreated controls. Twenty-four hours after treatment with chemical insults, cells were fixed in 4% paraformaldehyde for 20 min. The cells were then permeabilised and blocked with 0.2% Triton X-100% and 0.1% bovine serum albumin in PBS for 1 h. The cells were then incubated overnight at 4 °C with anti-beta-tubulin (1:1000, T8453, Sigma), tyrosine hydroxylase (1:1000, AB112, Abcam), and MAP2 (1:1000, AB92434, Abcam). The cells were washed 3 times for 5 min with PBS, then incubated with Alexa 555 anti-mouse (SAB4600066, Sigma) and/or Alexa 555 anti-rabbit (SAB4600069, Sigma) or Alexa 694 anti-rabbit secondary (A21449, Thermo) antibodies for 1 h at room temperature. After this, cells were incubated for 15 min in DAPI (1:1000) to label the nucleus of each cell. Labelled cells were imaged using EVOS microscopy equipment (M7000, Version 2) (Thermo Fisher, Bishop Meadow Road, Loughborough, LE11 5RE, UK) software. Fluorescence images were analysed using Fiji (ImageJ, version 2.9.0). Channels were separated, and a consistent threshold was applied to identify a positive signal in the red and blue channels. Double-positive cells were identified by co-localisation of red and blue signals using the “Image Calculator” (AND operation) to generate a co-localisation mask. Double-positive cells were manually counted by inspecting the co-localisation mask together with the original merged image across eight independent images per condition to avoid over- or under-counting. Counts were averaged per condition and normalised to the control condition (set to 100%) to allow comparison between groups.

LUHMES organoids, after MPP+ treatment, were removed from their six well plates and fixed in 4% paraformaldehyde overnight. After, they were washed with PBS 3 times for 5 min each and placed into 30% sucrose for a further 24 h. Once they sank to the bottom, they were embedded into OCT, immediately frozen on dry ice and placed into the −80 freezer for storage. The OCT blocks were sectioned using the cryostat (20 µM slices) and mounted onto glass cover slides. Once dry, the excess OCT was removed and using a hydrophobic pen a circle was drawn around each organoid. They were then permeabilised and blocked with 0.2% Triton X-100% and 0.1% bovine serum albumin in PBS for 1 h. The cells were then incubated overnight at 4° with anti-beta-tubulin (1:1000, T8453, Sigma), tyrosine hydroxylase (1:1000, AB112, Abcam), and MAP2 (1:1000, AB92434, Abcam). The cells were washed 3 times for 5 min with PBS, then incubated with Alexa 555 anti-mouse (SAB4600066, Sigma) and/or Alexa 555 anti-rabbit (SAB4600069, Sigma) secondary/or Alexa 694 anti-rabbit secondary (A21449, Thermo) antibodies for 1 h at room temperature. After this, cells were incubated for 15 min in DAPI (1:1000) to label the nucleus of each cell. Once washed and dried, gold anti-fade was added to each slide and a glass coverslip placed on top. Slides were allowed to dry for 48 h before storage at 4 °C. Slides were imaged using the confocal microscope. Images were then analysed using FIJI software (Version 2.9.0) as above.

### 2.5 RNA Extraction

Total RNA was extracted from cultured cells using TRIzol reagent (Invitrogen) following a modified manufacturer’s protocol. Cells grown in T75 flasks were lysed in 500 µL of TRIzol per well, homogenised by pipetting up and down, transferred to 1.5 mL tubes, vortexed briefly, and incubated on ice for 5 min. Chloroform: isoamyl alcohol (100 µL per 500 µL TRIzol) was added, mixed gently until uniform, and incubated on ice for 5–10 min. Phase separation was achieved by centrifugation at 15,000 × g for 15 min at 4 °C. The aqueous layer was carefully transferred to a new tube, avoiding the interphase. RNA was precipitated by adding cold isopropanol (500 µL per 1 mL TRIzol used), gently inverted, and incubated at room temperature for 15 min, followed by centrifugation at 15,000 × g for 15 min at 4 °C. The RNA pellet was washed with 80% ethanol, centrifuged twice to remove residual ethanol, air-dried for 5–10 min, and resuspended in 20 µL RNase-free water. RNA concentration and purity were determined using a Nanodrop 2000 spectrophotometer by measuring absorbance at 260 and 280 nm. For organoids, four organoids were added to a 1.5 mL Eppendorf, washed with PBS and then 500ul Trizol added. All other steps were the same.

### 2.6 cDNA synthesis and quantitative PCR (qPCR)

500 ng of RNA was used for cDNA synthesis. Reverse transcription was performed using an Invitrogen kit according to the manufacturer’s protocol. The reaction mixture (20 µL total volume) included reverse transcriptase, RNase inhibitor, random hexamers, dNTPs, buffer, and RNA template. The thermal cycling conditions were 65 °C for 10 min, 42 °C for 60 min, and 4 °C hold to terminate the reaction. Synthesised cDNA was stored at −20 °C until use. Quantitative PCR was carried out using SYBR™ Green Master Mix (Applied Biosystems) on a Quant Studio 5 Real-Time PCR System. Each 5 µL reaction contained 2.5 µL of diluted cDNA, 2.5 µL of SYBR Green master mix, and 0.5 µL each of forward and reverse primers. The cycling conditions were 95 °C for 2 min (initial denaturation), followed by 40 cycles of 95 °C for 15 s and 60 °C for 1 min. Melt curve analysis was performed to confirm the specificity of amplification. All reactions were run in technical triplicates. Gene expression was normalised to a housekeeping gene (B2M), and relative quantification was performed using the ΔΔCt method.

### 2.7 Superoxide production

MitoSOX Red (Invitrogen, Waltham, Massachusetts, U.S, 5 μM in DMSO) was used to detect mitochondrial superoxide. Cells were seeded in 12-well plates on glass coverslips at 125 × 10^3^ cells per well. Twenty-four hours after adhesion, both cell types underwent specific differentiation protocols. Cells were treated with 0.25 mM MPP+ and 24 hours after treatment, media were removed and MitoSOX Red (5 μM) diluted in the respective culture medium were added for 15 min. After this, cells were incubated for 15 min with DAPI (1:1000 in culture medium) to label the nucleus of each cell. Cells were immediately imaged at 20× on EVOS microscopy equipment and software. Images were then analysed using ImageJ software (Version 2.9.0).

In the case of 3D organoids, 24 hours after MPP+ treatment, media were removed and MitoSOX Red (5 μM) diluted in advanced DMEM/F12 was added for 15 min. After this, cells were incubated for 15 min with DAPI (1:1000 in culture medium) to label the nucleus of each cell. Once completed, they were placed in 4% PFA for 24 h. The PFA was removed, the organoids were washed with PBS 3 times (5 min each) and placed in sucrose for a further 24 h. Then, they were embedded in OCT and immediately frozen on dry ice and stored in −80 °C freezer. Using a cryostat, the OCT blocks were sectioned (20 μM) and placed onto glass slides to prepare for imaging on the confocal microscope. Images were then analysed using FIJI software (Version 2.9.0). Channels were separated, and a consistent threshold was applied to identify a positive signal in the red and blue channels. Double-positive cells, defined by co-localisation of red and blue signals, were manually counted across eight independent images. Counts were averaged per condition and normalised to the control condition (set to 100%) to allow comparison between groups.

### 2.8 Multi-electrode array (MEA) electrophysiology

Microelectrode arrays (60MEA200/30iR/T, Multi-Channel Systems MCS GmbH, 72770 Reutlingen, Germany) recorded neuronal activity. Briefly, microelectrode arrays were coated with 1 mL 50 μg/mL poly-L-ornithine (PLO; Sigma-Aldrich) and 1 μg/mL fibronectin from human plasma (Sigma-Aldrich) for 24 h at 37 °C. After removing the coating solution, arrays were air-dried for 10 min in the cell culture hood before 1 × 10^6^ LUHMES cells were added in 20 μL droplets to the centre of each MEA and placed in the cell culture incubator for 1 h. Arrays were then flooded with 1 mL of differentiation medium. Cells were differentiated for 4 days before measuring neuronal activity across multiple days. MEA recordings were analysed using the Neuromatic plugin (Rothman and Silver, 2018) on Igor Pro 8 (Wavemetrics, Version 8).

In 3D organoids microelectrode arrays were coated with 1 mL 50 μg/mL poly-L-ornithine (PLO; Sigma-Aldrich) and 1 μg/mL fibronectin from human plasma (Sigma-Aldrich) for 24 h at 37 °C. After removing the coating solution, arrays were air-dried for 10 min in the cell culture hood before the organoid was placed in the centre of the electrodes in 20 μL of media and placed into the incubator for 1 h. A further 20 μL of media was placed on top of the organoid alongside sterile water in the petri dish to prevent or reduce evaporation. Recordings were taken 24 h after treatment. MEA recordings were analysed using the Neuromatic plugin (Rothman and Silver, 2018) on Igor Pro 8 (Wavemetrics, Version 8).

### 2.9 Calcium imaging

Cells were seeded on glass coverslips in 12-well plates. After 24 h, the media was changed to differentiation media. Cells were then treated with MPP+ for 24 h. After this, the medium was removed, and cells were washed with artificial cerebrospinal fluid (ACSF) and replaced with Fluo-4-AM diluted in DMEM for 12 min. Cells were washed with ACSF and incubated in DMEM for 15 min before imaging. Glass coverslips were mounted onto the calcium chamber, flooded with ACSF, and placed on the microscope. Occular software (version 2.0) controlled the camera, and 5 min recordings were taken, including 2 min of baseline, and stimulated with ATP, GABA, Glutamic Acid and KCL. Recordings were then analysed using Sarfia plugin (Dorostkar, Dreosti, Odermatt and Lagnado, 2010) and Igor Pro 8 (Wavemetrics). Organoids (3 at a time) were placed into 400 μL of Advanced DMEM/F12 with Fluo-4-AM for 12 min. After organoids are placed into fresh Advanced DMEM/F12 for a further 15 min before imaging, Glass coverslips were mounted onto the calcium chamber, flooded with ACSF, the organoid added in and placed on the microscope. Occular software (version 2.0) controlled the camera, and 5 min recordings were taken, including 1 min of baseline, and 1 min stimulated with 0.1 mM ATP, 100 mM Glutamic acid and 50 mM KCL.

### 2.10 Statistical analysis

Statistical analysis was performed using GraphPad Prism (version 6, GraphPad Software, Inc., San Diego, CA, USA). Each experiment was performed in triplicate, and values are expressed as the mean ± standard deviation (SD) or ±SEM. Statistical significance is denoted as **** for *p* < 0.0001.

## 3 Results

### 3.1 3D LUHMES organoids’ exhibit enhanced neuronal network maturity compared to 2D cultures

We established baseline characteristics of 2D and 3D LUHMES culture systems, by first assessing the expression of synapsin, a presynaptic vesicle protein essential for neurotransmitter release and a marker of synaptic maturity (Sudhof, 2004). Immunofluorescence analysis revealed that 3D organoids express 8 times as much synapsin in comparison to 2D cultures (Figure 1). Quantification demonstrated that 3D organoids contained, on average 84% synapsin-positive cells, compared to only 9% in 2D cultures (p < 0.0001; Figure 1C). In 2D cultures, synapsin staining appeared sparse and punctate along isolated neurites, consistent with limited synaptic connectivity in planar configurations. In contrast, 3D organoids displayed dense, uniform synapsin expression throughout the structure, surrounding a compact cellular core and extending into peripheral neuritic regions (Figures 1A,B).

**FIGURE 1:**
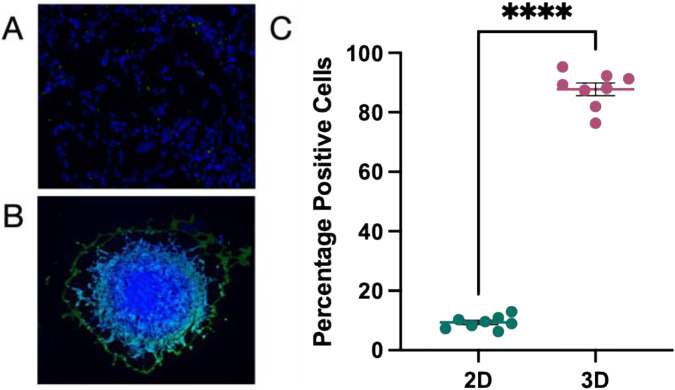
Representative images of Synapsin staining in LUHMES 2D and 3D cultures. **(A)** 2D and **(B)** 3D organoid cultures stained for nuclei (blue, DAPI) and synapsin (green). **(C)** Quantification of green: blue positive cells show significantly elevated levels in 3D cultures compared to 2D, supporting a more superior physiological relevance of the 3D model. (Data are shown as mean ± SEM. Statistical significance was determined using an unpaired t-test. ****p < 0.0001. n = 8).

This spatial organisation suggests the development of interconnected neuronal networks with extensive synaptic contacts, reflecting enhanced structural maturation in the three-dimensional environment. These findings establish that 3D LUHMES organoids achieve a higher degree of neuronal network complexity and synaptic development compared to traditional 2D cultures, providing a more physiologically relevant avenue for modelling neuronal function and degeneration.

### 3.2 MPP+ exposure significantly reduces viability and ATP production in both 2D and 3D LUHMES cultures, with enhanced sensitivity in 3D organoids

To model mitochondrial dysfunction, a characteristic of PD, we exposed both 2D and 3D LUHMES cultures to 0.25 mM MPP+ for 24 h and assessed cell viability. Both culture systems exhibited significant reductions in viability following MPP+ treatment (Figure 2). 2D cultures showed a 31% reduction in cell viability (*p* < 0.0001). While 3D organoids demonstrated a more pronounced decline by 35% (p < 0.0001). To determine whether reduced viability reflected mitochondrial dysfunction, we quantified intracellular ATP levels. Consistent with viability measurements, ATP production was significantly impaired in both models following MPP+ exposure (Figure 2B). 2D LUHMES cultures exhibited a 31% reduction in ATP levels (p < 0.0001), while 3D organoids showed a 36% decrease (p < 0.0001). Again, 3D organoids demonstrated significantly greater ATP depletion compared to 2D cultures (p < 0.05).

**FIGURE 2:**
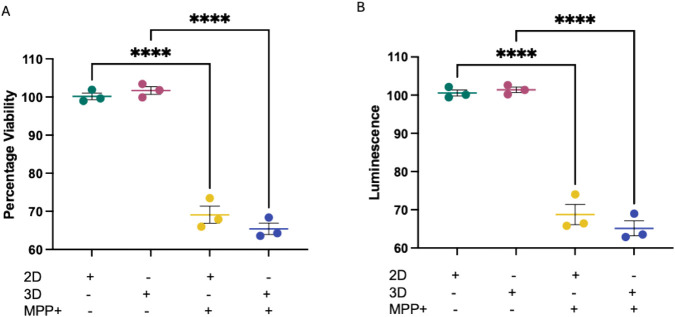
Effect of MPP+ on cell viability and ATP production. **(A)** Cell viability in 2D and 3D models **(B)** ATP production in 2D and 3D models. Both viability and ATP production are reduced after exposure to MPP+ in both the 2D and 3D models. Data are presented as means ± SEM, ****p < 0.0001, n = 3).

These results confirm that MPP+ successfully induces mitochondrial Complex I inhibition in both culture systems, leading to energy failure and cellular stress. Importantly, the enhanced sensitivity of 3D organoids suggests that the three-dimensional architecture, with its increased metabolic demands and potential oxygen/nutrient gradients, may more accurately recapitulate the vulnerability of dopaminergic neurons *in vivo*.

### 3.3 Exposure to MPP+ differentially impairs dopaminergic and neuronal markers in both 2D and 3D LUHMES cultures

To assess the impact of MPP+ on dopaminergic neuronal identity and structural integrity, we performed immunofluorescence analysis of tyrosine hydroxylase (TH, the rate-limiting enzyme in dopamine synthesis), MAP2 (a dendritic marker of mature neurons), and βIII-tubulin (TUJ1, a marker of neurite outgrowth and general neuronal cytoskeleton).

In 2D LUHMES cultures, MPP^+^ treatment resulted in significant reductions in all three markers (Figure 3). TH+ cells decreased by 60% (*p* < 0.0001, Figure 3B). This dramatic loss of TH expression indicates either dedifferentiation of dopaminergic phenotype or selective death of TH-expressing neurons. MAP2 expression also declined significantly, by approx. 54% (p < 0.0001, Figure 3F). This suggests structural deterioration of dendritic compartments, consistent with early neurodegenerative changes. Interestingly, TUJ1 expression showed more modest effects, declining by approx. 20% (p < 0.01, Figure 3E). The relative preservation of TUJ1 compared to TH and MAP2 may indicate that general cytoskeletal elements remain partially intact even as more specialized dopaminergic and dendritic markers are lost.

**FIGURE 3:**
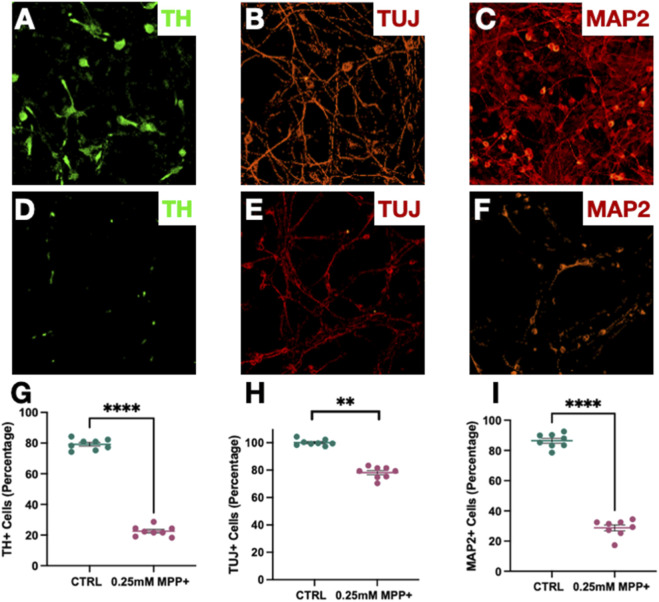
Acute MPP+ Exposure reduces TH, TUJ and MAP2 expression in 2D LUHMES cells. **(A–D)** Representative images showing Tyrosine Hydroxylase (TH) without **(A)** and with **(D)** _MPP+, **(B–E)** TUJ1 (βIII-Tubulin) without **(B)** and with **(E)**_MPP+, and **(C–F)** MAP2 expression with **(F)** and without **(C)** _MPP+ in LUHMES cells following 24-h MPP^+^ treatment. **(G–I)** Quantification of expression of TH, TUL and MAP2 across 2D LUHMES Cells. Scale bar for each image is 150uM. Data are presented as means ± SEM, ****p < 0.0001, n = 8).

In 3D LUHMES organoids, MPP+ exposure resulted in severe and uniform impairment across all markers (Figures 4A–D). TH expression decreased by 60% (p < 0.0001, Figure 4D) comparable to 2D cultures. However, both MAP2 and TUJ1 showed dramatically greater sensitivity in the 3D cultures. MAP2-positive cells declined by 70% (p < 0.0001, Figure 4F), substantially more than the 54% reduction observed in 2D cultures. This enhanced MAP2 loss in 3D organoids suggests more extensive dendritic network collapse, potentially reflecting the disruption of complex three-dimensional neuritic architectures that cannot form in 2D. TUJ1 expression in 3D organoids fell by 62% (p < 0.0001, Figure 4C). This contrasts sharply with the 20% reduction in 2D cultures and indicates that the general neuronal cytoskeleton is far more vulnerable in the 3D context. This differential vulnerability may result from the increased mechanical stresses, metabolic demands, or intercellular dependencies present in three-dimensional neuronal networks. Qualitatively, MPP+-treated 3D organoids exhibited not only reduced marker expression but also spatial disorganization, with loss of the characteristic dense core structure and fragmentation of peripheral neuritic projections (Figure 4).

**FIGURE 4:**
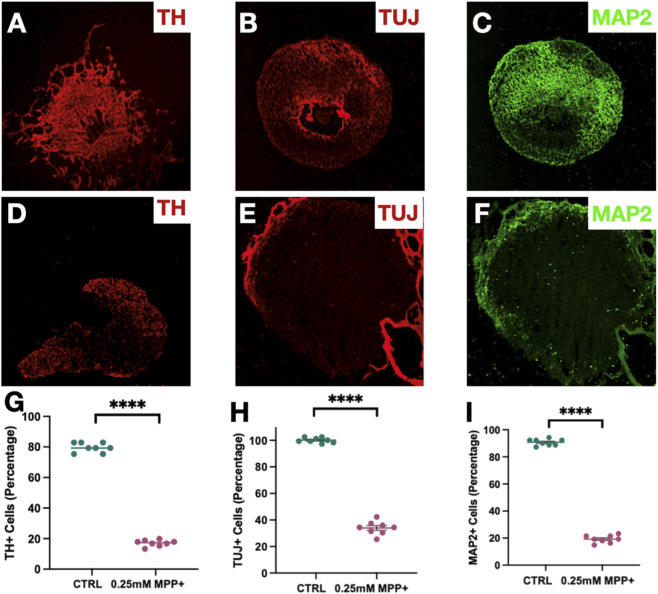
MPP+ exposure reduces TH, TUJ and MAP2 expression in 3D LUHMES cells. **(A–D)** Representative images showing Tyrosine Hydroxylase (TH) without **(A)** and with **(D)** _MPP+, **(B–E)** TUJ1 (βIII-Tubulin) without **(B)** and with **(E)**_MPP+, and **(C–F)** MAP2 expression with **(F)** and without **(C)** _MPP+ in LUHMES cells following 24-h MPP^+^ treatment. **(G–I)** Quantification of expression of TH, TUJ and MAP2 across 3D LUHMES Cells. Data are presented as means ± SEM, ****p < 0.0001, n = 8).

qPCR analysis was also carried out to confirm both the neuronal and dopaminergic expression of discrete genes (*SLC6A3* (DAT), *TH* (TH) and *MAP2* (MAP2)) in both 2D and 3D models and their sensitivity in chemically induced PD. MPP+ exposure significantly reduced expression levels of all three genes in both the 2D and 3D model (Table 1).

**TABLE 1:** Effect of MPP+ treatment on gene expression in 2D and 3D models. MPP+ exposure reduces TH, DAT and MAP2 gene expression in 2D and 3D LUHMES cells.

Gene	Model	Control (FC ± SEM)	Treatment (FC ± SEM)
DAT	2D	0.189 ± 0.088	0.01 ± 0.003
	3D	1 ± 0.515	0 ± 0
TH	2D	1 ± 0.12	0.034 ± 0.018
	3D	0.145 ± 0.108	0 ± 0
MAP2	2D	1 ± 0.278	0.397 ± 0.148
	3D	0.421 ± 0.188	0.432 ± 0.224

### 3.4 MPP+ exposure induces reactive oxygen species production in both 2D and 3D LUHMES cultures

Mitochondrial dysfunction caused by Complex I inhibition typically results in electron leak and reactive oxygen species (ROS) generation, a key contributor to oxidative stress in PD. To assess ROS production, we used MitoSOX Red, a mitochondria-targeted fluorescent probe selective for superoxide in response to 0.25 mM MPP^+^ for 24 h (Figure 5). In 2D LUHMES cultures, control cells showed minimal baseline MitoSOX fluorescence (15% ROS-positive cells). Following MPP+ treatment, ROS-positive cells increased to 80% (p < 0.0001, Figures 5A,B) an approximately 5-fold increase. Importantly, ROS accumulation appeared heterogeneous across the 2D culture, with some cells showing intense perinuclear staining while neighbouring cells displayed minimal signal (Figure 5A). This heterogeneity likely reflects cell-to-cell variation in mitochondrial health, metabolic state, or antioxidant capacity, with individual cells responding asynchronously to the toxic insult.

**FIGURE 5:**
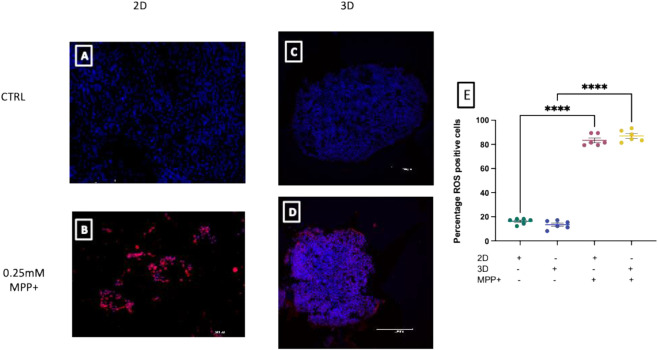
MitoSOX expression in 2D and 3D LUHMES cells following MPP+ exposure. **(A,B)** Representative images of the effect of MPP+ on MitoSOX in 2D (**(A)** (0 mM), **(B)** (0.25 mM)) and 3D (**(C)** (0 mM), **(D)** (0.25 mM)) LUHMES cell models. **(E)** Quantification of MitoSOX in 2D and 3D LUHMES cells. Data are presented as means ± SEM, ****p < 0.0001, n = 6).

In 3D LUHMES organoids, control conditions similarly showed low baseline ROS (15% positive cells). MPP+ treatment increased ROS-positive cells to 85% (p < 0.0001, Figures 5A,B) a 6-fold increase that was marginally but significantly higher than in 2D cultures (p < 0.05). ROS distribution in 3D organoids was uniform, with consistent MitoSOX staining throughout the organoid structure (Figure 5C). This homogeneous response contrasts sharply with the patchy 2D pattern and suggests that oxidative stress propagates more uniformly through interconnected three-dimensional neuronal networks.

### 3.5 3D LUHMES cultures display enhanced electrophysiological activity at baseline but greater functional impairment following MPP+ exposure

To assess functional neuronal network activity, we employed multi-electrode array (MEA) recordings. This approach enables quantification of network-level properties including mean firing rate (MFR), spike amplitude, and action potential kinetics. Under basal conditions, 3D LUHMES organoids exhibited substantially higher spontaneous electrical activity compared to 2D cultures (Figure 6D). Mean firing rate in 3D organoids reached, on average, 0.72 Hz, compared to, on average, 0.61 Hz in 2D cultures (p < 0.0001). This 1.2-fold increase in baseline activity correlates with the enhanced synaptic connectivity and network maturation observed in synapsin staining (Figure 1). Additionally, spike amplitudes were significantly larger in 3D organoids (74.3 pV) compared to 2D cultures (52.7 pV) (p < 0.0001, Figure 6D). Larger spike amplitudes can indicate stronger synaptic inputs, more synchronized network activity, or improved electrode-tissue coupling in the 3D configuration. Together, these baseline findings confirm that 3D LUHMES organoids develop more sophisticated electrophysiological properties than conventional 2D cultures.

**FIGURE 6:**
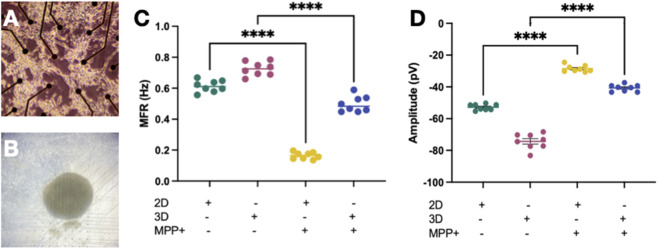
Multi-electrode array recording of LUHMES in 2D and 3D cultures, treated with 0.25 mM MPP+. **(A,B)** Representative images of 2D **(A)** and 3D **(B)** cultures on MEA **(C)** Calculated Amplitude and mean firing rate (MFR) across the different models. **(D)** Mean spike amplitudes across the models. Data are presented as means ± SEM, ****p < 0.0001, n = 8).

Following 24-h MPP+ exposure, both culture systems showed significant reductions in network activity (Figure 6D). While reduction in network activity was the desired outcome after MPP+ exposure, it is important that while we see impact, we do not go beyond the therapeutic window of no return. In 2D cultures, the mean firing rate decreased by on average 65% (p < 0.0001). Spike amplitude declined by 46% (p < 0.0001). In contrast, 3D organoids exhibited a mean firing rate reduction of by 32% (p < 0.0001) and a spike amplitude reduction of 40%. So, while the 2D models exhibited a more sensitive response to the MPP+, we believe these cells are impaired beyond the point of no return, with limited possibilities to understand the therapeutic benefit on network regeneration.

### 3.6 Exposure to MPP+ impairs calcium signalling dynamics with differential effects in 2D and 3D LUHMES cultures

Intracellular calcium signalling is central to neuronal function, regulating neurotransmitter release, synaptic plasticity, and cell survival. To assess calcium signalling integrity, we performed live-cell calcium imaging using Fluo-4 AM and quantified both spontaneous calcium activity and responses to pharmacological stimuli, including ATP (purinergic receptor agonist), glutamate (excitatory neurotransmitter), and GABA (inhibitory neurotransmitter). Under control conditions, both 2D and 3D LUHMES cultures exhibited robust baseline (spontaneous) calcium oscillations, with 3D organoids showing moderately higher baseline activity (Figures 7, 8A). Peak calcium responses (ΔF/F) in 2D cultures were not significantly different from 3D cultures (Figure 8A). However, 3D organoids displayed more coordinated calcium waves propagating across cells, whereas 2D cultures showed more sporadic, cell-autonomous activity (visible in representative images, Figure 7). Following acute MPP+ treatment (0.25 mM, 24 h), baseline calcium activity was significantly reduced in both models (Figure 8A). In 2D cultures, baseline peak ΔF/F decreased by 10% (p < 0.05). In 3D organoids, baseline activity fell more dramatically by around 60% (p < 0.0001), indicating a more complete loss of spontaneous calcium oscillations in the three-dimensional network.

**FIGURE 7:**
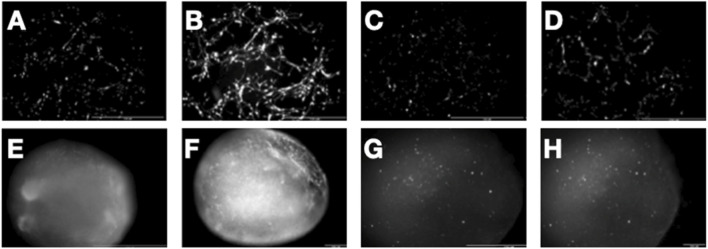
Representative images of 2D and 3D LUIHMES cells in response to Flou-4AM dye and stimulation with ATP. **(A,B)** 2D LUHMES control, baseline and ATP stimulated **(C,D)** 2D LUHMES MPP+ treated,, baseline and ATP stimulated **(E,F)** 3D LUHMES control,, baseline and ATP stimulated **(G,H)** 3D LUHMES MPP+ treated at baseline and ATP stimulated.

**FIGURE 8:**
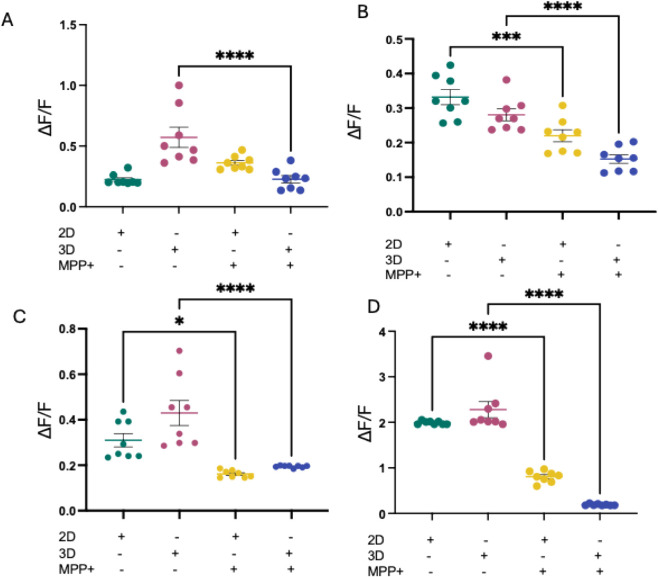
Peak Calcium Response of LUHMES cells exposed to MPP+ (0.25 mM). **(A)** Baseline peak responses, **(B)** Glutamic Acid, **(C)** ATP and **(D)** GABA response in 2D and 3D LUHMES cells after MPP+ treatment. Data are presented as means ± SEM, ****p < 0.0001, n = 8).

Stimulus-evoked calcium responses showed similar patterns of impairment. In control 2D and 3D cultures the glutamate response was reduced by 30% in the presence of MPP+ (p < 0.01). Similarly, in 2D cultures, ATP elicited robust calcium increases (Figure 8C). MPP+ treatment reduced this by 35% (p < 0.001). In 3D organoids, control ATP responses were similar but fell dramatically by 52% following MPP+ (p < 0.0001). GABA also elicited a calcium response in 2D that was inhibited by MPP+ (50% reduction, p < 0.0001). Again, 3D organoids GABA responses were reduced by nearly 100% with MPP+ treatment (p < 0.0001). To assess not only peak responses but also the sustained calcium signalling over time, we quantified area under the curve (AUC) for each stimulus condition (Figure 9). AUC analysis largely aligns with the peak response findings but revealed additional insights into the temporal dynamics of calcium dysregulation. Baseline activity (Figure 9A), in 2D cultures showed modest AUC reductions following MPP+ (30%, p < 0.0001), while 3D organoids exhibited near-complete elimination of sustained baseline activity (70%, p < 0.0001). , with all other stimulus showing similar trends in reduction to the above peak calcium responses. However, it is worth noting that MPP+ exposure to the 3D organoids seems to have a more pronounced effect on 3D organoids that 2D monolayers (Figure 9).

**FIGURE 9:**
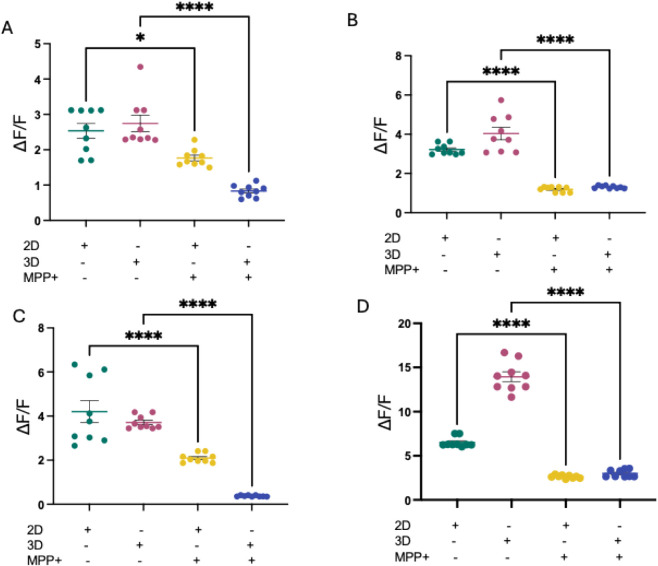
AUC Calcium Response of LUHMES cells exposed to MPP+ (0.25 mM). **(A)** Baseline peak **(B)** Glutamic Acid**, (C)** ATP and **(D)** GABA response in 2D and 3D LUHMES cells after MPP+ treatment. Data are presented as means ± SEM, ****p < 0.0001, n = 8).

## 4 Discussion

Our data highlights that LUHMES-derived 3D organoids provide a more physiologically relevant platform for modelling dopaminergic neurodegeneration than conventional two-dimensional cultures. We demonstrate that 3D organoids exhibit enhanced neuronal maturation, greater sensitivity to mitochondrial toxins, and more developed functional network properties, making them a valuable tool for mechanistic investigation and drug discovery in Parkinson’s disease research.

We observed a substantial increase in synapsin expression in 3D LUHMES organoids (9-fold higher than 2D cultures), providing strong evidence for enhanced synaptic development and neuronal network maturation in three-dimensional culture environments (Figure 1). Synapsin, a phosphoprotein associated with synaptic vesicles, plays essential roles in neurotransmitter release, synaptic plasticity, and maintenance of vesicle pools (Sudhof, 2004). Its abundance in LUHMES 3D organoids suggests the formation of extensive synaptic contacts and functional neuronal circuits that cannot develop in planar 2D configurations. The enhanced maturation likely results from multiple factors inherent to 3D culture. First, 3D environments permit cells to establish contacts in all spatial directions, dramatically increasing the opportunities for synapse formation compared to the limited contact surfaces available in monolayers (Pamies et al., 2017). Second, three-dimensional architectures generate microenvironmental gradients of oxygen, nutrients, and secreted factors that more closely approximate *in vivo* conditions and can influence neuronal differentiation and maturation (Qian, Song and Ming, 2019). Third, mechanical properties differ between 2D substrates (stiff plastic and glass in our studies) and 3D organoids (softer, more brain-like), with substrate stiffness known to influence neuronal development and function (Saha et al., 2008).

The baseline electrophysiological properties of 3D organoids further support their enhanced maturation. The 1.5-fold higher spontaneous firing rate and larger spike amplitudes indicate development of more extensive synaptic networks with greater excitability and improved synchronisation. These properties align with developmental maturation of neuronal networks, where increasing connectivity and synaptic strength drive higher spontaneous activity (Blankenship and Feller, 2010). Previous studies of iPSC-derived cortical organoids similarly reported enhanced electrophysiological maturation in 3D compared to 2D cultures, including development of oscillatory network activity resembling *in vivo* brain dynamics (Trujillo et al., 2019).

A central finding of this study is that 3D LUHMES organoids exhibit sensitivity to MPP^+^ -induced mitochondrial dysfunction across multiple endpoints. This differential vulnerability provides important insights into how cellular architecture may influence responses to neurodegenerative insults. 3D neuronal networks exhibit higher baseline metabolic activity than 2D cultures, as evidenced by their increased spontaneous firing rates. This elevated activity creates greater dependence on mitochondrial ATP production. When Complex I is inhibited by MPP+, the energy deficit in 3D organoids may be more severe over time because cells cannot meet the elevated ATP demands required to maintain ion gradients, synaptic transmission, and normal cellular processes. In contrast, 2D cultures with lower baseline activity may tolerate partial ATP depletion better because their energy requirements are more modest.

This aligns with our viability and ATP data, which show that 3D organoids exhibit greater ATP depletion than 2D cultures (36% vs. 28%, Figure 2). While this difference in ATP production is only moderate, the functional consequences appear disproportionate, suggesting that 3D networks may be operating closer to a critical energetic threshold. Once ATP levels fall below this threshold, cascade failures occur as cells cannot maintain membrane potentials, calcium homeostasis, or synaptic function. These differential effects of MPP+ were also evident on TH, MAP2, and TUJ1 expression which provides insight into mechanisms of dopaminergic neuron loss. TH was severely reduced in both 2D (Figure 3) and 3D (Figure 4) systems (∼70%), consistent with its known sensitivity to mitochondrial stress and oxidative damage (Rausch, Wang and Radad, 2022). TH gene expression is regulated by numerous transcription factors sensitive to cellular stress, including NURR1 and PITX3, which may be downregulated following MPP+ exposure (Kadkhodaei et al., 2009). MAP2, a dendritic marker critical for microtubule stabilization and dendritic morphology, showed a reduction in 2D cultures (54%) but a more severe loss was observed in 3D organoids (70%). This difference likely reflects the more complex dendritic architecture present in 3D systems. In organoids, neurons develop elaborate networks with extensive branching patterns that depend on continuous energy supply and functional microtubule dynamics (Harris, Hogberg, Hartung and Smirnova, 2017; Lyu, DeMarco, Sweet and Grubisha, 2025; Zhang et al., 2014). When mitochondrial function fails, these energy-intensive structures cannot be maintained, leading to dendritic retraction and MAP2 degradation. In simpler 2D cultures with less complex dendritic architecture, the energetic burden is lower, permitting partial preservation. TUJ1 (βIII-tubulin) was relatively preserved in 2D cultures (only 20% reduction) but severely affected in 3D organoids (62% reduction). This dramatic difference suggests that the axonal cytoskeleton in 3D systems faces unique stresses absent in 2D cultures. In 3D culture, axons must navigate space, maintain long-distance connections, and participate in more extensive synaptic networks all of which are energy-intensive processes.

Reactive Oxygen Species (ROS) generation, measured via MitoSOX staining, was elevated by 80% (p-value 0.0001) in all LUHMES cultures treated with MPP^+^. This correlates with our ATP data and may suggest that prolonged mitochondrial inhibition not only depletes energy stores but also generates toxic oxidative byproducts that can lead to cell death and neuroinflammation (Figure 5) (Uttara, Singh, Zamboni, & Mahajan, 2009). The spatial patterns of ROS accumulation in 2D versus 3D cultures provide insights into how cellular architecture influences oxidative stress responses. In 2D cultures, MitoSOX staining appeared heterogeneous, with some cells showing intense signal while neighbours remained relatively unaffected. This pattern suggests that individual cells’ antioxidant capacity and mitochondrial health determine their vulnerability (Dewitt et al., 2006). In contrast, 3D organoids exhibited remarkably uniform ROS distribution throughout the entire structure. This homogeneous pattern could reflect several mechanisms: (1) propagation of oxidative stress through gap junction-mediated transfer of ROS or depleted antioxidants between connected cells, (2) coordinated metabolic responses across the network due to shared microenvironmental conditions, or (3) amplification of ROS production through inflammatory signalling cascades that coordinate cellular responses. This uniform oxidative stress response in 3D organoids better models the tissue-level oxidative damage observed in PD, where markers of oxidative stress are found throughout affected brain regions rather than in isolated cells (Dias, Junn and Mouradian, 2013). The ability to model these coordinated stress responses represents a significant advantage of 3D systems for understanding neurodegeneration.

There is a network-level vulnerability that is evident in our MEA and calcium imaging data (Figures 7–9). The near-complete loss of coordinated calcium waves and dramatic reduction in synchronous electrical activity in MPP+-treated 3D organoids suggest system-wide network failure, not just cellular dysfunction. In 2D cultures, by contrast, cells can function relatively independently, so some neurons retain activity even when neighbours are severely compromised.

The electrophysiological and calcium imaging data provide strong evidence for the added value of 3D LUHMES organoids. While biochemical and molecular markers are valuable, functional assessments ultimately determine whether neurons can perform their essential roles in neural circuits. Our MEA and calcium imaging results demonstrate that 3D organoids not only exhibit enhanced baseline function but also capture dimensions of functional failure that are potentially not as robust in 2D systems. Calcium signalling is essential for neurotransmitter release, synaptic plasticity, and neuronal survival. The inability to mount appropriate calcium responses to ATP, glutamate, and GABA indicates a fundamental failure of cellular excitability and signal transduction. Mechanistically, this likely reflects multiple deficits, including depleted intracellular ATP required for calcium pumps (SERCA, PMCA), impaired mitochondrial calcium buffering, altered expression or function of plasma membrane calcium channels, and potential endoplasmic reticulum stress (Bruce, 2010). The more severe calcium dysregulation in 3D organoids may result from their greater dependence on coordinated calcium signalling for network function.

Traditional rodent models of PD, while valuable for studying motor function and testing therapies *in vivo*, face significant limitations. Transgenic mice expressing PD-related mutations rarely develop substantial dopaminergic cell loss or robust Lewy body pathology (Blesa et al., 2012). Toxin models (MPTP, 6-OHDA) produce acute dopaminergic lesions but do not capture the chronic progressive nature of human PD. Moreover, species differences in dopamine metabolism, brain size, and aging timescales limit translational relevance. LUHMES organoids, being human-derived, avoid these cross-species translation issues while offering experimental advantages including speed, reproducibility, and ethical considerations. iPSC-derived dopaminergic neurons and midbrain organoids represent powerful patient-specific models, however, they face practical challenges including lengthy differentiation protocols (often 60–90 days or more), significant batch-to-batch variability, incomplete maturation, and high cost (Bono, Missale and Fiorentini, 2022). LUHMES-derived 3D organoids occupy a unique niche combining rapid differentiation, consistent dopaminergic phenotype, human origin, and enhanced 3D architecture. This combination makes them particularly suited for, (1) mechanistic studies of dopaminergic vulnerability requiring reproducible models, (2) toxicity screening where speed and throughput are essential, (3) preclinical drug testing where human-relevant responses are needed, and (4) fundamental neuroscience investigations of dopaminergic neuron biology in physiologically relevant contexts.

Despite their advantages, LUHMES-derived 3D organoids have important limitations. Current LUHMES organoids consist primarily of neurons derived from a single source, thus they lack the cellular diversity of the intact brain. Emerging evidence implicates glial cells, particularly microglia and astrocytes, as critical contributors to PD pathogenesis through neuroinflammation and impaired metabolic support (Kam, Hinkle, Dawson and Dawson, 2020). Future iterations should incorporate these cell types through co-culture approaches or by generating assembloids that combine LUHMES-derived neurons with glial organoids. In addition, LUHMES cells do not carry patient-specific mutations in LRRK2, SNCA, PINK1, or other PD-associated genes. LUHMES organoids lack blood vessels, limiting their size, longevity, and ability to model blood-brain barrier interactions. Vascularization strategies including co-culture with endothelial cells or transplantation into host animals are being developed for other organoid systems and could be applied to LUHMES organoids (Cakir et al., 2019). (While 3D LUHMES organoids show enhanced maturation compared to 2D cultures, they may still not fully recapitulate the complexity of adult human dopaminergic neurons in that they may lack certain region-specific markers or fail to develop Lewy body-like protein aggregations seen in aged patient-derived neurons. Finally LUHMES organoids do not recapitulate the anatomical organization of the nigrostriatal pathway or other brain circuits affected in PD. Assembloid approaches that combine midbrain-like LUHMES organoids with striatal organoids could model these circuit-level interactions (Miura et al., 2020).

## 5 Conclusion

We have developed and characterized three-dimensional LUHMES organoids as an enhanced model for dopaminergic neurodegeneration in Parkinson’s disease. These organoids exhibit superior neuronal maturation, enhanced synaptic connectivity compared to conventional 2D cultures. Critically, 3D organoids demonstrate greater sensitivity to mitochondrial toxins across molecular, cellular, and network-level endpoints, capturing dimensions of neurodegeneration that are often lost in 2D culture systems. These findings underscore the importance of developing and utilising physiologically relevant disease models and LUHMES-derived 3D organoids offer enhanced insight into disease progression, therapeutic response, and potential regenerative strategies. These models can be further refined by incorporating microglia, astrocytes, or vasculature, enhancing their fidelity.

## Data Availability

The original contributions presented in the study are included in the article/supplementary material, further inquiries can be directed to the corresponding author.
